# Hepatic Encephalopathy-Associated Cerebral Vasculopathy in Acute-on-Chronic Liver Failure: Alterations on Endothelial Factor Release and Influence on Cerebrovascular Function

**DOI:** 10.3389/fphys.2020.593371

**Published:** 2020-11-20

**Authors:** Laura Caracuel, Esther Sastre, María Callejo, Raquel Rodrigues-Díez, Ana B. García-Redondo, Isabel Prieto, Carlos Nieto, Mercedes Salaices, Ma Ángeles Aller, Jaime Arias, Javier Blanco-Rivero

**Affiliations:** ^1^Departamento de Fisiología, Facultad de Medicina, Universidad Autónoma de Madrid, Madrid, Spain; ^2^Instituto de Investigación Hospital Universitario La Paz, Madrid, Spain; ^3^Departamento de Farmacología y Terapéutica, Facultad de Medicina, Universidad Autónoma de Madrid, Madrid, Spain; ^4^Centro de Investigación Biomédica en Red de Enfermedades Cardiovasculares, Madrid, Spain; ^5^Departamento de Cirugía General y Digestiva, Hospital Universitario la Paz, Madrid, Spain; ^6^Departamento de Cirugía Cardiaca, Hospital Universitario la Paz, Madrid, Spain; ^7^Cátedra de Cirugía, Facultad de Medicina, Universidad Complutense de Madrid, Madrid, Spain

**Keywords:** acute-on-chronic liver failure, hepatic encephalopathy, cerebral vasculature, bradykinin, nitric oxide, prostaglandin I_2_

## Abstract

The acute-on-chronic liver failure (ACLF) is a syndrome characterized by liver decompensation, hepatic encephalopathy (HE) and high mortality. We aimed to determine the mechanisms implicated in the development of HE-associated cerebral vasculopathy in a microsurgical liver cholestasis (MHC) model of ACLF. Microsurgical liver cholestasis was induced by ligating and extracting the common bile duct and four bile ducts. Sham-operated and MHC rats were maintained for eight postoperative weeks Bradykinin-induced vasodilation was greater in middle cerebral arteries from MHC rats. Both Nω-Nitro-L-arginine methyl ester and indomethacin diminished bradykinin-induced vasodilation largely in arteries from MHC rats. Nitrite and prostaglandin (PG) F_1α_ releases were increased, whereas thromboxane (TX) B_2_ was not modified in arteries from MHC. Expressions of endothelial nitric oxide synthase (eNOS), inducible NOS, and cyclooxygenase (COX) 2 were augmented, and neuronal NOS (nNOS), COX-1, PGI_2_ synthase, and TXA_2_S were unmodified. Phosphorylation was augmented for eNOS and unmodified for nNOS. Altogether, these endothelial alterations might collaborate to increase brain blood flow in HE.

## Introduction

Liver cholestasis is a well-known clinical syndrome that may be induced by multiple pathologies (Rodríguez-Garay, 2003). Independently of its origin, liver cholestasis is clinically characterized by jaundice, discolored urine, pale stools, pruritus, spleen enlargement, liver cirrhosis, hepatorenal syndrome, and portal hypertension, the latter due to an increase in portal vein resistance (Boyer, 2007). Alterations in endothelium-derived vasoactive factors are determinant in the circulatory disturbances observed in liver cholestasis, leading to the development of portal hypertension and to a splanchnic and systemic vasodilation (Pateron et al., 2000; Iwakiri and Groszmann, 2007). Different experimental models of liver cholestasis have shown a decompensation after six postoperative weeks, together with hepatic encephalopathy (HE) and ascites, leading to an acute-on-chronic liver failure (ACLF; García-Moreno et al., 2005; Gilsanz et al., 2017). This decompensation can aggravate these cardiovascular disturbances, causing hypotension, decreased effective blood volume, and increased cardiac output (Møller et al., 2001; Sastre et al., 2016b; Caracuel et al., 2019), eventually leading to patient death.

Hepatic encephalopathy is a complex neuropsychiatric syndrome present in around 30% of the patients with ACLF (Lizardi-Cervera et al., 2003). Although insufficient clearance of toxins from blood, hyperammonemia, increased oxidative stress and enhanced inflammatory pathways are pivotal in the pathophysiology of HE, abnormalities in cerebral blood flow are also implicated in the development of this syndrome (Shawcross et al., 2004; Shawcross and Jalan, 2005). Both increases and decreases in cerebral vasculature resistance have been reported in HE, depending on the seriousness of the pathology, and can lead to either a decrease or an increase in cerebral blood flow, respectively (Guevara et al., 1998; Hollingsworth et al., 2010; Sunil et al., 2012; Dam et al., 2013; Zheng et al., 2013). In fact, an increased cerebral blood flow has been reported to correlate with raised intracranial pressure in patients with ACLF (Kerbert et al., 2017).

Several factors modulate physiological regulation of cerebral blood flow, including neural, metabolic and endothelial factors. Focusing on the last, endothelium exerts a profound influence on blood flow in cerebral arteries by releasing nitric oxide (NO) and prostanoids, among other factors (Andresen et al., 2006; Peterson et al., 2011), thus regulating the tone of underlying smooth muscle. Under inflammatory conditions, the role of vascular endothelium can be modified in cerebral vessels (Hernanz et al., 2004; Maccarrone et al., 2017), leading to increases in NO and prostanoids release.

Despite the reports regarding modifications in cerebral blood flow in HE, to the best of our knowledge the studies concerning possible endothelial modifications in cerebral vasculature in ACLF are scarce. Thus, the objective of this study was to determine the mechanisms implicated in the development of cerebral vasculopathy in microsurgical liver cholestasis (MHC), a model of ACLF, which develops HE (García-Moreno et al., 2005; Gilsanz et al., 2017). Specifically, we analyzed whether MHC modifies the endothelial function in rat middle cerebral artery (MCA), and the possible alterations of the endothelial vasoactive factors in cerebral vasculature.

## Materials and Methods

### Ethical Statements

All experimental procedures were approved by the Ethical Committee of the Universidad Autónoma de Madrid, and the Comunidad de Madrid (PROEX 322/16), are in compliance with NIH guidelines, with the ARRIVE guidelines (Animal Research: Reporting *in Vivo* Experiments) for how to REPORT animal experiments, and follow the European Parliament Directive 2010/63/EU guidelines.

### Animals

Thirty-four male Wistar rats (Initial weight: 337.9 ± 6.91 g) were purchased from Harlan Ibérica SL, Barcelona, Spain and housed in the Animal Facility of the Universidad Autónoma de Madrid (Registration number EX-021U). Rats were held in groups of 2 in appropriate cages in controlled environmental conditions (20–24°C, 55% relative humidity and 12 h light-dark cycle). All rats had access to drinking water and specific rat chow *ad libitum*.

Animals were randomly divided into two groups: Sham-operated (SO; *n* = 17), in which the common bile duct was dissected; and MHC (*n* = 17), in which the extrahepatic biliary tract was resected (García-Moreno et al., 2005; Sastre et al., 2016b; Gilsanz et al., 2017; Caracuel et al., 2019). Surgery was performed under aseptic but not sterile conditions. In summary, rats were anesthetized with Ketamine hydrochloride (100 mg/kg) and xylazine (12 mg/kg) i.m. In the SO group, the bile duct and its lobular branches were dissected. In the MHC group, the extrahepatic bile tract was resected using a binocular operatory microscope (Zeiss, OPMI 1-FR). First, the common bile duct was ligated (silk 4/0) and sectioned close to the beginning of its intrapancreatic portion. Dissection and sectioning between ligatures of all biliary branches that drain the hepatic lobes is possible using a binocular operatory microscope (Zeiss, OPMI 1-FR). The dissection and excision of the bile ducts from the four liver lobes of the rat was done without injuring either the portal and/or, most especially, the arterial vascularization of these lobes. The abdomen was closed in two layers by continuous running sutures using an absorbable suture (3/0 polyglycolic acid) and silk (3/0). Buprenorphine s.c. (0.05 mg/kg/8 h) was administered postoperatively for analgaesia the first 24 h after the surgery.

### Systolic Blood Pressure

Systolic blood pressure (SBP) was measured using the tail-cuff method 8 weeks after the surgery was performed (Xavier et al., 2010; Sastre et al., 2016b; Caracuel et al., 2019).

### Portal Vein Pressure Measurement

After an overnight fasting, splenic pulp pressure, an indirect measurement of portal pressure (PP) was measured in four animals from each group, by inserting a fluid filled 20-gauge needle into the splenic parenchyma (Sastre et al., 2016b; Caracuel et al., 2019). The needle was joined to a PE-50 tube, and then connected to a pressure recorder (PowerLab 200 ML 201) and to a transducer (Sensonor SN-844) with a Chart V 4.0 computer program (ADI Instruments), that was calibrated before each experiment. The pressure reading was considered satisfactory when a stable recording was produced and respiratory variations were observed. Previous studies have demonstrated the excellent correlation between splenic pulp pressure and PP (Kravetz et al., 1986). These animals were discarded for the following experiments.

### Animal Euthanasia and Sample Collection

After an overnight fasting, animals were sacrificed by CO_2_ inhalation followed by decapitation. Ascitic liquid was carefully extracted to measure its volume. Liver and spleen were extracted, rinsed in saline solution and weighed. Kidneys were extracted, rinsed in saline buffer, and frozen in liquid nitrogen and stored at −70°C, until use. Brain was removed and placed in cold Krebs–Henseleit solution (KHS, in mmol/L: NaCl 115; CaCl_2_ 2.5; KCl 4.6; KH_2_PO_4_ 1.2; MgSO_4_⋅7H_2_O 1.2; NaHCO_3_ 25; glucose 11.1, Na_2_ EDTA 0.03) at 4°C. Cerebral arteries were dissected under a stereoscopic magnifying glass (Euromex Holland, Barcelona).

### Gene Expression Studies

Total RNA was isolated from 1/4 rat renal samples with TriPure Isolation Reagent (Sigma). cDNA was synthesized using the NZY First-Strand cDNA Synthesis Kit (Nzytech) using 2 μg of total RNA, following the manufacturer’s instructions. Real Time PCR was performed in 7500 Fast ABI System (Life Technologies Inc.) to detect gene expression of Rat NGAL (FW: 5′-GAGCGATTCGTCAGCTTTGC-3′; Rv:5′ATTGGTCGGTGGGAACAGAG-3′) and Rat KIM-1 (FW:5′-AAGCCGAGCAAACATTAGTGC-3′; RV:5′-TGAGCTAGAATTCAGCCACACA-3′). mRNA copy numbers were calculated for each sample by the instrument software using Ct value (“arithmetic fit point analysis for the lightcycler”). Results are expressed in copy numbers, calculated relative to control rat, after normalization with rat β2 microglobulin (Fw: 5′ ACCGTGATCTTTCTGGTGCTTG-3′; Rv: 5′ TAGCAGTTG AGGAAGTTGGGCT-3′).

### Vascular Reactivity Experiments

For vascular reactivity experiments, we used MCA segments of 2 mm in length (internal diameter: SO: 231.4 ± 1.8 μm, (*n* = 6); MHC: 233.8 ± 1.4 μm (*n* = 4); *P* > 0.05) were mounted in a wire myograph for measurement of isometric tension according to a described method (Hernanz et al., 2004). Segment contractility was tested by an initial exposure to a high-KCl solution (120 mmol/L KCl-KHS), observing a similar vasoconstriction in MCA segments from both SO and MHC animals (in mN/mm: SO: 2.216 ± 0.65; MHC: 2.194 ± 0.38; *P* > 0.05).

Endothelium integrity was determined by the ability of 1 μmol/L bradykinin (BK) to relax segments precontracted with 10 μmol/L 5-hydroxytryptamine (5HT, serotonin). Afterward, concentration-response curves for BK (0.1 nmol/L to 10 μmol/L) were performed in 5HT-precontracted MCA segments from SO and MHC rats. The effects of the non-selective NO synthase inhibitor N^ω^ -nitro-L-arginine methyl ester (L-NAME, 0.1 mmol/L) or the non-specific cyclooxygenase (COX) inhibitor indomethacin (10 μmol/L) on the concentration-response curves for BK were investigated. The inhibitors were added 30 min before the concentration-response curve was performed. The inhibitors did not alter the arterial basal tone.

The vasodilator response to the NO donor, diethylamine NON-Oate, (DEA-NO, 0.1 nmol/L–0.1 mmol/L) was determined in 5HT-precontracted MCA segments from both experimental groups.

### NADPH Oxidase Activity

The specific superoxide anion production generated by NADPH oxidase activity was determined as previously described (Llévenes et al., 2018). For that purpose, frozen cerebral vasculature was homogenized in an ice-cold buffer containing 20 mmol/L KH_2_PO_4_, 1 mmol/L EGTA and 150 mmol/L sucrose. The reaction was started by the addition of a lucigenin (5 μmol/L)/NADPH (100 μmol/L) mixture to the tissue homogenate. Chemiluminescence was determined every 2.4 s for 5 min in a plate luminometer (AutoLumat LB 953, Berthold, Germany). Buffer blank was subtracted from each reading. Luminescence was normalized by protein concentration, and data were expressed as chemiluminiscence units/μg protein.

### Vasoactive Factor Production

The production of NO, TXA_2_ and PGI_2_ were measured using the commercial kits Nitric Oxide Assay Kit (Abcam Laboratories), Thromboxane (TX) B_2_ ELISA kit (Cayman Chemical) and 6-keto Prostaglandin (PG) F_1α_ ELISA Kit (Cayman Chemical), respectively. For sample collecting, the cerebral vasculature was pre-incubated for 30 min in 2 mL of KHS at 37°C, continuously gassed with a 95% O_2_–5% CO_2_ mixture (stabilization period). This was followed by two washout periods of 10 min in a bath of 0.2 mL of KHS. Then, the medium was collected to measure basal release. Afterward, arteries were subjected to 10 μmol/L 5-HT for 2 min, and then a BK concentration curve (0.1 nmol/L to 10 μmol/L) was applied at 1 min intervals. Afterward, the medium was collected to measure the BK-induced vasoactive factor release. For NO production measurement, some samples were collected in presence of 5 μmol/L L-NPA (specific neuronal nitric oxide synthase – nNOS-inhibitor), 1 μmol 1400W (specific inducible NOS – iNOS-inhibitor) or 0.1 mmol/L L-NAME. The inhibitors were added 30 min before the concentration-response curve to BK was performed. Samples were immediately frozen in liquid nitrogen and conserved at −70°C until the assays were performed. The different assays were carried out according to the manufacturers’ instructions.

### Western Blot Analysis

For Western blot analysis, the cerebral vasculature were homogenized in a buffer composed of 1 mmol/L sodium vanadate (a protease inhibitor), 1% SDS, and 0.01 mol/L pH 7.4 Tris–HCl. Protein content was determined using a DC^TM^ Protein Assay (Bio-Rad Laboratories, Hercules, CA, United States). Homogenates containing 30 μg protein were electrophoretically separated on a 7.5% (iNOS, eNOS, PS1177–eNOS, nNOS, and PS1417 nNOS), or 10% (COX-1, COX-2, TXA_2_ synthase, and PGI_2_ synthase) SDS–polyacrylamide gel (SDS–PAGE), and then transferred to polyvinyl difluoride membranes (Bio Rad Immun-Blot w) overnight at 4°C, 230 mA, using a Bio-Rad Mini Protean Tetra system (Bio-Rad Laboratories, Hercules, CA, United States) containing 25 mmol/L Tris, 190 mmol/L glycine, 20% methanol, and 0.05% SDS. PageRuler Prestained Protein Ladder (Thermo Scientific) was used as molecular mass markers. The membranes were blocked for 3 h at room temperature in a Tris-buffered-saline solution (100 mmol/L, 0.9% w/v NaCl, 0.1% SDS) with 5% bovine serum albumin before being incubated overnight at 4°C with mouse monoclonal anti-eNOS (1:1000, Transduction Laboratories), rabbit polyclonal anti-PS1177 eNOS (1:1000, Abcam laboratories), mouse monoclonal anti-nNOS (1:1000, Transduction Laboratories), rabbit polyclonal anti-PS1417 nNOS (1:1000, Abcam laboratories); mouse monoclonal anti-iNOS (1:1000, Transduction Laboratories); rabbit monoclonal anti-COX-1 (1:1000 dilution, Abcam laboratories); rabbit polyclonal anti-COX-2 (1:2000 dilution, Cayman Chemical); rabbit polyclonal anti-TXA_2_ synthase (1:500 dilution, Cayman Chemical); or rabbit polyclonal anti-PGI_2_ synthase (1:500 dilution, Cayman Chemical). After washing, the membrane was incubated with a 1:2000 dilution of the appropriate secondary antibody (anti-mouse or anti-rabbit immunoglobulin G antibody) conjugated to horseradish peroxidase (GE Healthcare, Little Chalfont, United Kingdom). The membrane was thoroughly washed and the immunocomplexes were detected using an enhanced horseradish peroxidase/luminol chemiluminescence system (ECL Plus, GE Healthcare, Little Chalfont, United Kingdom). Finally, the images were developed and quantified using the Quantity One software (v. 4.6.6, Biorad, Spain). The same membranes were used to determine β-actin expression (1:50000 dilution, Sigma-Aldrich, Spain) and the content of the latter was used to correct protein expression.

### Drugs and Solutions

Drugs used were 5-hydroxytryptamine creatinine sulfate (serotonin), bradykinin, sodium vanadate, L-NAME (N^ω^ -nitro-l-arginine methyl ester), indomethacin, SDS, Trizma-base and bovine serum albumin (Sigma-Aldrich; Spain). Stock solutions (10 mmol/L) were made in bidistilled water and kept at −20°C. Appropriate dilutions were made in KHS on the day of the experiment.

### Data Analysis

Graph representation and statistical analysis were performed using GraphPad Prism 8.0 software (CA, United States). The relaxations induced by BK were expressed as a percentage of the initial contraction elicited by 5HT (in mN: SO: 1.18 + 0.15; MHC: 1.29 + 0.19, *P* > 0.05). Non-lineal regressions were performed. Areas under the curve (AUC) were calculated from the individual concentration-response plots. Results were expressed as mean ± standard error of the mean (SEM). In vascular reactivity experiments, statistical analysis was performed by means of two-way analysis of variance (ANOVA). For differences of AUC (dAUC), vasoactive substance release and Western blot experiments, the ROUT method was used to identify and remove outliers. Moreover, a Shapiro–Wilk test was applied to confirm the normality of the population data, followed by a Student’s *t*-test statistical analysis. *P* < 0.05 was considered significant.

## Results

### Animal Evolution

**FIGURE 1:**
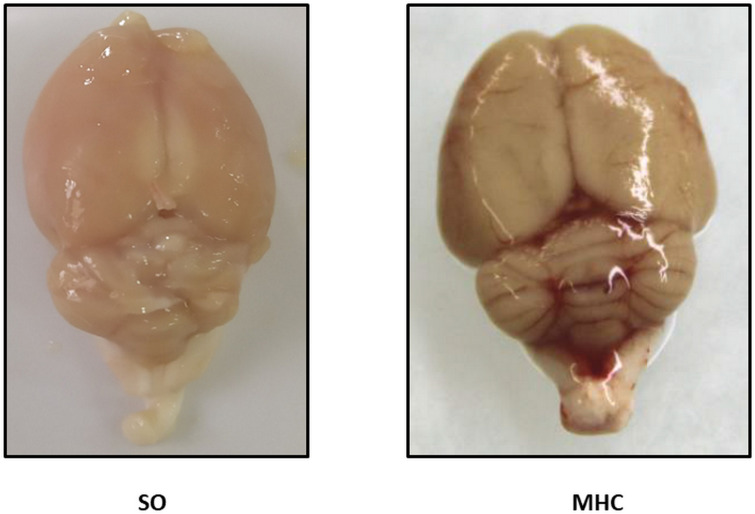
Representative pictures showing the brains from Sham-Operated (SO) and rats submitted to microsurgical liver cholestasis (MHC).

All MHC animals showed jaundice and choluria. Paraesophageal, splenorenal and pararectal collateral vessels developed in MHC animals (Data not shown). Final body weight and body weight gain were lower in MHC animals. Systolic blood pressure was lower, and PP was enhanced in the MHC group. Moreover, both spleen and liver hypertrophy, and extravasation of ascitic fluid were present in MHC animals (Table 1). We also found that both neutrophil gelatinase-associated lipocalin (NGAL), and Kidney injury molecule 1 (KIM-1), renal damage markers, were enhanced after MHC (Table 2). In addition, a jaundice-derived color and an inflammatory phenotype were found in rats submitted to MHC (Figure 1). Altogether, these results confirm that MHC is an appropriate experimental model for studying hepatic and extrahepatic complications of this pathology.

**TABLE 1:** Effect of microsurgical liver cholestasis (MHC) on body weight (BW), body weight gain (BWG), systolic blood pressure (SBP), portal pressure (PP), liver weight (LW), spleen weight (SW) and ascitic liquid extravasation in Wistar rats.

	BW (g)	BWG (g)	SBP (mm Hg)	PP (mm Hg)	LW (g)	SW (g)	Ascitic liquid (mL)
SO	420.0 ± 9.98	77.12 ± 9.51	120.0 ± 3.67	8.49 ± 0.14	13.54 ± 0.51	0.74 ± 0.04	–
MHC	341.8 ± 12.42*	8.85 ± 5.28*	79.76 ± 3.31*	16.07 ± 1.38*	22.41 ± 1.73*	1.68 ± 0.12*	15.84 ± 5.69

Results are expressed as means ± SEM. * P < 0.05 versus SO (Student t-test). n = 4–17 animals each group.

**TABLE 2:** NGAL and KIM-1 levels in kidneys from Sham-Operated (SO) and microsurgical liver cholestasis (MHC) rats.

	NGAL (n-fold)	KIM-1 (n-fold)
SO	1 ± 0.21	1 ± 0.38
MHC	2.309 ± 0.67*	2.098 ± 0.52*

Results are expressed as means ± SEM. * P < 0.05 versus SO (Student t-test). n = 6–9 animals each group.

### Bradykinin-Induced Vasodilation

**FIGURE 2:**
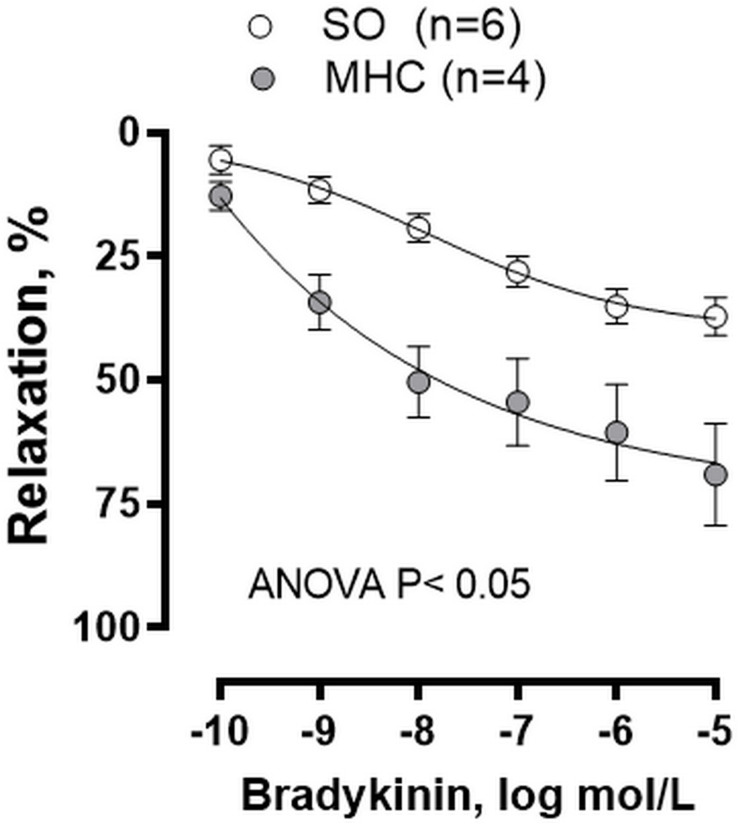
Effect of microsurgical liver cholestasis (MHC) on the concentration-dependent relaxation to bradykinin in rat middle cerebral arteries. Sham-Operated (SO): *n* = 6; MHC: *n* = 4. Results (mean ± SEM) were expressed as a percentage of the initial contraction elicited by 5HT.

Large cerebral arteries, like MCA, strongly contribute to total cerebrovascular resistance (Faraci and Heistad, 1990, 1998). For that reason, we analyzed whether MHC could modify the endothelium dependent BK-induced response in MCA. We observed that BK produced a concentration-dependent vasodilator response in MCA from both groups, which was greater in MHC animals (Figure 2).

### Role of Endothelium-Derived NO on the Vasodilator Response to BK

**FIGURE 3:**
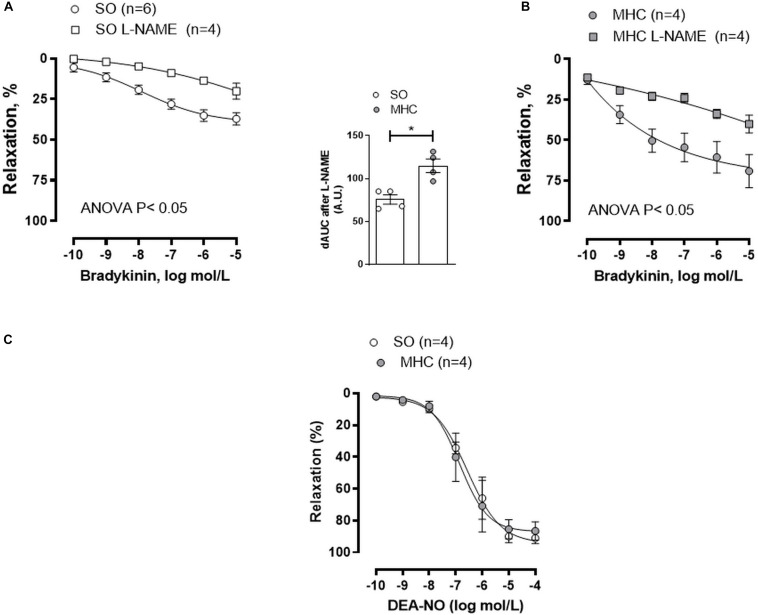
Effect of preincubation with NOS inhibitor L-NAME on the concentration-dependent relaxation to bradykinin in rat middle cerebral arteries from Sham-Operated (SO), **(A)**; (*n* = 4–6 segments from different animals) and microsurgical liver cholestasic (MHC), **(B)**; (*n* = 4 segments from different animals) rats. Results (mean ± SEM) were expressed as a percentage of the initial contraction elicited by 5HT. The attached graph shows the differences of area under the curve (dAUC) to bradykinin for segments in the absence or presence of 0.1 mmol/L L-NAME. dAUC values are expressed as a percentage of the difference of the corresponding AUC for the segments in the absence of L-NAME. **(C)** Vasodilator response to NO donor DEA-NO in arteries from SO and MHC rats (*n* = 4 segments from different animals in each group). Results (mean ± SEM) were expressed as a percentage of the initial contraction elicited by 5HT.

One of the pivotal endothelial vasoactive factors modified by inflammation is NO, which exerts a vasodilator effect in cerebral vessels (Faraci and Heistad, 1998; Andresen et al., 2006). To determine a different role of NO in SO and MHC animals, we preincubated MCA segments with the non-selective NO synthase inhibitor L-NAME. This drug diminished BK-induced vasodilation in MCA segments from both groups, being this decrease greater in arteries from rats submitted to MHCcerebral vasculature from MHC (Figures 3A,B, and dAUC). It is also remarkable that the vasodilator role to NO donor DEA-NO was similar in MCA segments from both experimental groups (Figure 3C). This result rules out possible differences in smooth muscle sensitivity to NO in our experimental conditions.

**FIGURE 4:**
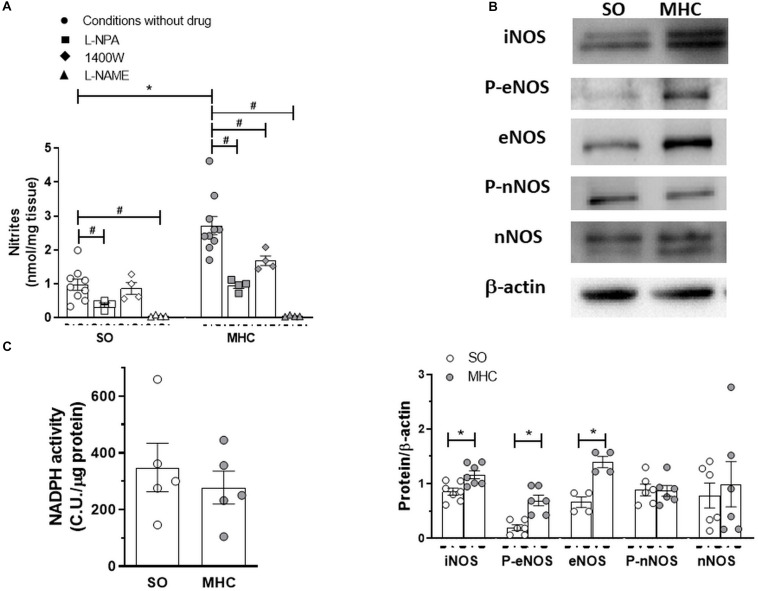
**(A)** Bradykinin-induced nitrite release in cerebral vasculature from Sham-Operated (SO; *n* = 4-9 animals) and microsurgical liver cholestasic (MHC; *n* = 4–10 animals) rats. Effect of preincubation with specific nNOS inhibitor L-NPA, specific iNOS inhibitor 1400W, or non-specific NOS inhibitor L-NAME. Data (Mean ± SEM) were expressed as nmol nitrites/mg tissue. *P < 0.05 SO vs MHC (Student *t*-test). ^#^*P* < 0.05 conditions without drug vs. conditiosmn with drug (Student *t*-test). **(B)** Western blot analysis for and inducible nitric oxide synthase (iNOS), total and phosphorylated endothelial nitric oxide synthase (eNOS) in the Ser 1177 residue (P-eNOS), and total and phosphorylated neuronal nitric oxide synthase (nNOS) in the Ser 1417 residue (P-nNOS), in cerebral arteries from Sham-Operated (SO) and microsurgical liver cholestasis (MHC) rats. The blots are representative of 4–7 segments from each group. Lower panels show densitometry analysis for the expression of each protein. Results (mean ± SEM) were expressed as the relation between the signal obtained for the protein analyzed and the signal obtained for β-actin. *P < 0.05 SO vs MHC (Student t test). **(C)** NADPH activity in cerebral vessels from SO and MHC rats (*n* = 5 different animals from each group). Results (mean ± SEM) were expressed as chemiluminiscence units (CU.)/μg protein.

To analyze whether this differential role of NO was due to NO release, we analyzed the release of the stable NO metabolite nitrite, finding that BK-induced nitrite release was greater in the cerebral vasculature from MHC animals (Figure 4A).

Multiple enzymes are implicated in the NO synthesis. When analyzing the influence of different NOS inhibitors in nitrite release, we found that the specific nNOS inhibitor L-NPA diminished BK-induced nitrite release in a similar extent in cerebral vasculature from both experimental groups (in percentage of inhibition: SO: 61.3 + 6.5; MHC: 65.1 + 3.1; *P* > 0.05). In addition, the specific iNOS inhibitor 1400W significantly decreased BK-induced nitrite release only in arteries from MHC rats, while the non-specific NOS inhibitor L-NAME abolished this release in the cerebral vasculature from both SO and MHC rats (Figure 4A). We also observed an increase in iNOS expression in arteries from MHC rats. Moreover, both eNOS expression and phosphorylation in Ser 1177 were enhanced in MHC cerebral arteries, while neither nNOS expression nor Ser1417 phosphorylation were modified by MHC (Figure 4B).

Aside from NO release, it is also important to remark that NO function also depends on its bioavailability. Liver pathologies enhance oxidative stress in different tissues, including vascular tissue (Xavier et al., 2010), thereby reducing NO function. The fact that NADPH oxidase activity, the main producer of superoxide anion, was similar in cerebral vasculature from both experimental rules (Figure 4C), allowed us to rule out possible alterations in NO bioavailability.

### Role of COX-Derived Prostanoids on the Vasodilator Response to BK

**FIGURE 5:**
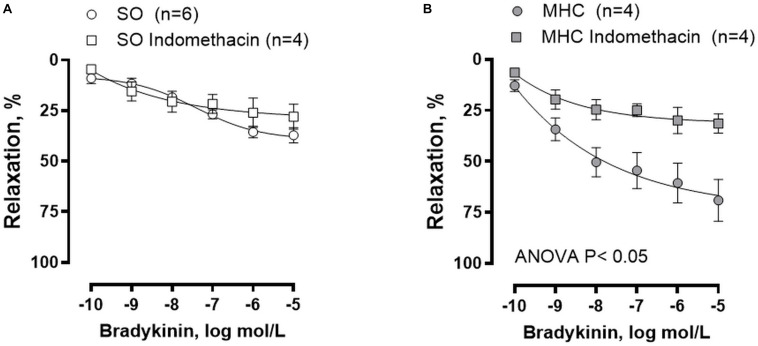
Effect of preincubation with COX inhibitor indomethacin on the concentration-dependent relaxation to bradykinin in rat middle cerebral arteries from Sham-Operated (SO), **(A)**; (*n* = 4–6 segments from different animals) and microsurgical liver cholestasic (MHC), **(B)**; (*n* = 4 segments from different animals) rats. Results (mean ± SEM) were expressed as a percentage of the initial contraction elicited by 5HT.

Modifications of other vasoactive factors could also be involved in the pathogenesis of arterial vasodilation in MHC (Theodorakis et al., 2003). To analyze the possible participation of prostanoids in the enhanced BK-induced vasodilation observed in MHC rats, we preincubated MCA with the unspecific COX inhibitor indomethacin. We observed that BK-induced vasodilation was not modified by indomethacin in MCA from SO animals, while it was diminished in arteries from MHC rats (Figures 5A,B).

**FIGURE 6:**
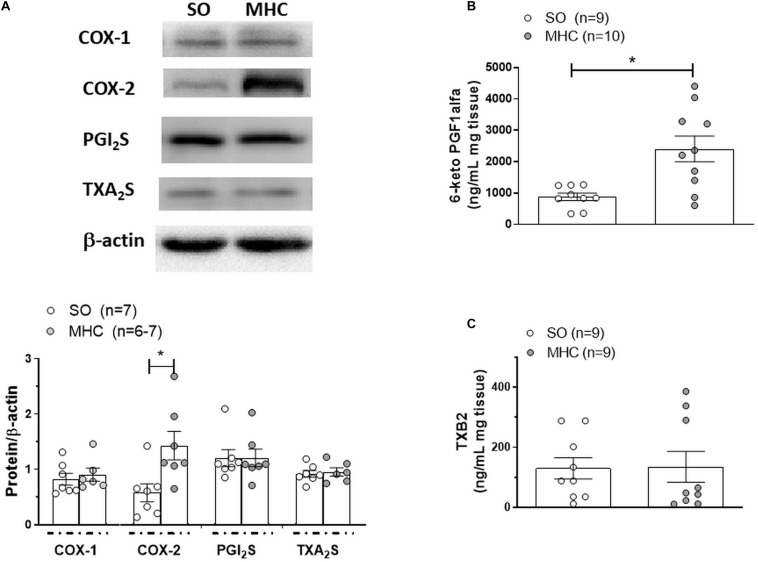
**(A)** Western blot analysis for cyclooxygenase (COX) 1 and 2, PGI_2_ synthase (PGI_2_S) and TXA_2_ synthase (TXA_2_S) in cerebral arteries from Sham-Operated (SO) and microsurgical liver cholestasis (MHC) rats. The blots are representative of 6–7 segments from each group. Lower panels show densitometry analysis for the expression of each protein. Results (mean ±SEM) were expressed as the relation between the signal obtained for the protein analyzed and the signal obtained for β-actin. *P < 0.05 SO vs MHC (Student *t*-test). Effect of microsurgical liver cholestasis (MHC) on cerebral artery 6-Keto PGF_1α_ release **(B)**; (SO: *n* = 9 animals; MHC: *n* = 10 animals) and TXB_2_ release **(C)**; (n = 10 animals from each experimental group) Results (mean ± SEM) were expressed as pg prostanoid/mL mg tissue. *P < 0.05 SO vs MHC (Student *t*-test).

An overexpression of the enzymes implicated in prostanoid synthesis has been reported in different vascular beds in rats with liver pathologies (Xavier et al., 2010). When analyzing COX-1, we observed a similar expression in cerebral arteries from both experimental groups, while the expression of COX-2 was greater in cerebral arteries from MHC animals (Figure 6A).

Among the prostanoids implicated in the regulation of vascular tone, both vasodilator PGI_2_ and vasoconstrictor TXA_2_ have a relevant role in cerebral vessels (Andresen et al., 2006; Peterson et al., 2011; Maccarrone et al., 2017). When analyzing the expressions of PGI_2_ synthase and TXA_2_ synthase, the enzymes implicated in their synthesis, we observed no differences among groups (Figure 6A). Moreover, when analyzing 6-keto PGF1α and TXB_2_, the respective stable metabolites of PGI_2_ and TXA_2_, we observed that MCH enhanced BK-induced 6-keto PGF_1α_ release but did not modify BK-induced TXB_2_ release (Figures 6B,C).

## Discussion

The present study analyses the alterations in the cerebrovascular function in rats submitted to MHC, a model of ACLF, which develops HE. The results obtained show an enhanced endothelium-dependent BK-induced vasodilation in MHC rats, which is due to increases in the vasodilator factors NO and PGI_2_ release.

Extrahepatic cholestasis is the most common model to study the vascular complications of obstructive liver cholestasis. Rats with MHC develop ACLF symptoms such as hepatomegaly, PH, enlarged spleen, collateral portosystemic circulation, and ascites, as reported earlier (Aller et al., 2012; Sastre et al., 2016b; Caracuel et al., 2019). It is also remarkable that we previously described alterations in albumin, total protein, bilirubin, and transaminases after this experimental procedure (Gilsanz et al., 2017; Caracuel et al., 2019). We can also highlight that these animals developed hepatorenal syndrome, since both the renal damage markers NGAL and KIM-1, were enhanced in kidneys from MHC rats. In is also interesting to remark the jaundice-derived color and the inflammatory phenotype that we observed in brains from rats submitted to MHC. In addition, a previous study from our group showed alterations in different brain structures, such as the hippocampus, several weeks after this surgery was performed (García-Moreno et al., 2002). These alterations were accentuated after 8 weeks of the surgery (Braissant et al., 2019), consequently developing behavioral changes including both cognitive and motor impairment, which worsen together with the severity of the pathology (García-Moreno et al., 2002; Giménez-Garzó et al., 2015; Braissant et al., 2019). Altogether, these data allow us to confirm that MHC is an appropriate experimental model for studying hepatic and extrahepatic complications, including HE alterations, developed in ACLF.

Microsurgical liver cholestasis is characterized by the development of systemic vascular complications, which can be the origin of the HE associated to ACLF (Jalan and Williams, 2002; Wright et al., 2014). Moreover, previous studies have reported that a systemic proinflammatory state can activate microglia and consequently produce a neuroinflammatory situation, therefore worsening the neuropsychiatric symptoms of HE (Butterworth, 2013; Rama Rao et al., 2014; Macías-Rodríguez et al., 2015). This proinflammatory state can modify the release of endothelial vasoactive factors in several vascular beds, including cerebral vessels (Faraci and Heistad, 1998; Andresen et al., 2006), consequently altering cerebral blood flow and vascular resistance, and contributing to the development of the brain abnormalities observed in this pathology. Large cerebral arteries, like MCA, strongly contribute to total cerebrovascular resistance, being the main determinants of local microvascular pressure (Faraci and Heistad, 1990, 1998). For that reason, we analyzed whether MHC could modify the endothelium dependent BK-induced response in MCA. We observed an augmented BK vasodilation in vessels from MHC rats, as has been reported in other vascular beds (Xavier et al., 2010; Hollenberg and Waldman, 2016; Caracuel et al., 2019). As we commented in the introduction section, alterations in the balance of vasodilator and vasoconstrictor agents are frequent in liver pathologies, leading to blood flow alterations in multiple vascular beds (Xavier et al., 2010; Bolognesi et al., 2014; Bosch et al., 2015; Sastre et al., 2016b; Caracuel et al., 2019). One of the pivotal endothelial vasoactive factors modified by inflammation is NO, which exerts a vasodilator effect in cerebral vessels both *in vivo* and *ex vivo* by activating soluble guanylate cyclase and/or producing smooth muscle hyperpolarization through potassium channel opening (Faraci and Heistad, 1998; Andresen et al., 2006). A dysregulation of NO production is a common denominator of most of the symptoms accompanying liver pathologies. The role of NO is reported to vary in different vascular beds. Thus, a decrease in NO levels has been described in portal vein, increasing the resistance in this vascular bed. Conversely, increases in NO have also been reported in systemic vasculature and plasma, where they participate in the development of hyperdynamic circulation in splanchnic and systemic circulation (Bolognesi et al., 2014; Bosch et al., 2015; Hollenberg and Waldman, 2016). We observed that, similar to earlier reports in different splanchnic and systemic vascular beds (Xavier et al., 2010; Sastre et al., 2016b; Caracuel et al., 2019), BK-induced nitrite (the stable NO metabolite) levels were significantly increased in cerebral arteries from MHC rats compared to SO animals. This result correlates with the fact that preincubation with unspecific NOS inhibitor L-NAME diminished BK-induced relaxation to a greater extent in MCA from MHC rats, hence confirming a major functional role for NO in this experimental group. In addition, the fact that the vasodilator role to NO donor DEA-NO was similar in MCA segments from both experimental groups allowed us to rule out possible differences in both smooth muscle sensitivity to NO and in the NO signaling pathway in MCA due to MHC.

NO can be synthetized through the action of both constitutive eNOS and nNOS, and inducible iNOS. The increase in iNOS expression in splanchnic vasculature suggests involvement by iNOS-derived NO in the development of hyperdynamic circulation in different liver pathologies (Bhimani et al., 2003; Ferguson et al., 2006; Xavier et al., 2010). Given the fact that iNOS plays a relevant role in NO synthesis in brain under inflammatory conditions (Hernanz et al., 2004; Rama Rao et al., 2014), we analyzed possible differences in iNOS expression under our experimental conditions, finding an increase in this enzyme expression in cerebral arteries from MHC animals. Consequently, the increase in iNOS expression in MHC animals would explain the augmented NO release observed in this experimental group. The fact that the specific iNOS inhibitor 1400W reduced nitrite release only in cerebral vessels from MHC rats corroborates this hypothesis, thereby confirming the relevant role of iNOS in the vascular alterations in MHC.

We must also take into account that both constitutive isoforms eNOS and nNOS are also present in vascular endothelium of cerebral arteries (Andresen et al., 2006; Iwakiri and Groszmann, 2007). An increase in eNOS expression was described in the splanchnic and systemic vascular beds in several liver pathology models (Wright et al., 2012; Lin et al., 2014), and eNOS-derived NO has been described to be high in cerebral vessels under inflammatory status (Ando et al., 2004; Maccarrone et al., 2017). Thus, we cannot rule out possible alterations in eNOS expression/activation in cerebral arteries from MHC animals. An increase in eNOS expression was found in cerebral vessels from MHC rats. Additionally, we observed an augmented eNOS phosphorylation at Ser1177, indicating enhanced eNOS activation, as we previously described in mesenteric vascular bed (Xavier et al., 2010; Caracuel et al., 2019).

Regarding nNOS-derived NO, it is implicated in the increased vasodilation observed in splanchnic and systemic vasculature in liver pathologies, including ACLF (Xu et al., 2000; Sastre et al., 2016b), while several reports have described that nNOS is present in cultured endothelial cells from different vascular beds, exerting an anti-inflammatory role (Chakrabarti et al., 2012). We found no modifications in either nNOS expression or phosphorylation on Ser1417. Moreover, the specific inhibition of nNOS diminished NO release in a similar extent in both experimental groups. These results contrast with these studies previously mentioned. The different tissues and experimental model could explain this discrepancy. Altogether, these results show that the observed increase in NO release in cerebral arteries from MHC rats could be due to augmented eNOS and iNOS activity.

Multiple studies have shown that MHC induced a systemic increase of oxidative stress. Specially, enhanced pro-oxidative biomarkers and diminished antioxidant mechanisms have been reported in brain from MHC rats (Ommati et al., 2020). In blood vessels, superoxide anions can modulate the role of NO, diminishing its bioavailability. Although there are several sources of reactive oxygen species, the enzyme NADPH oxidase is the main producer of the excessive superoxide anions in vascular tissue (Miller et al., 2010; Sastre et al., 2016a; Llévenes et al., 2018). When analyzing the activity of this enzyme, we found no differences between our experimental groups. This result might contrast with the fact that liver pathologies enhance vascular oxidative stress in other vascular beds (Xavier et al., 2010), while we also found no differences in superoxide anions in superior mesenteric artery from MHC rats (unpublished results from our group). It is interesting to remark that an enhanced systemic oxidative stress systemic does not necessary correlate with local alterations in superoxide anions (Torres et al., 2019; Llévenes et al., 2020). In addition, we cannot rule out the participation of other sources of vascular superoxide anions. Since the functional analysis of NO-induced vasodilation showed no differences between SO and MHC rats, we can infer that the oxidative stress participation in cerebral vasculature might not change due to MHC.

Aside from excess NO generation in the splanchnic circulation, data from eNOS and iNOS knockout mice suggests that modifications of vasoactive factors other than NO could be involved in the pathogenesis of arterial vasodilation in liver pathologies (Theodorakis et al., 2003). Endothelial prostanoids, synthetized through COX activation, participate in the regulation of vascular tone in healthy situations, depending on the vascular bed analyzed, but their production can be modified under certain inflammatory pathological situations (Brian et al., 2001; Hernanz et al., 2004; Mollace et al., 2005; Blanco-Rivero et al., 2007; Peterson et al., 2011; Wiggers et al., 2016). In fact, multiple studies have already focused on the role of COX-derived vasodilator (PGI_2_) and vasoconstrictor (TXA_2_) prostanoids in the vascular disturbances observed in liver pathologies (Xavier et al., 2010; Macías-Rodríguez et al., 2015; Caracuel et al., 2019). Therefore, we aimed to determine the possible differential influence of prostanoids on the BK-induced vasodilation in cerebral vessels from SO and MHC rats. For that purpose, we incubated MCA segments with the non-specific COX inhibitor indomethacin, observing that this drug exerted no influence in vessels from SO animals, as previously described (Wiggers et al., 2016), while it diminished BK-induced vasodilation in arteries from MHC animals. Previously, an overexpression of COX-2 was described in splanchnic vasculature in liver pathologies (Xavier et al., 2010). What is more, alterations in constitutive COX-1 expression have also been reported in several tissues, including endothelium and brain tissue (Zheng et al., 2013; Kerbert et al., 2017). Therefore, we aimed to investigate possible alterations in the expression of both COX isoforms. We found a greater COX-2 expression in cerebral arteries from MHC rats, while COX-1 was not modified in our experimental conditions. Therefore, the increase in COX-2 expression we observed in cerebral arteries suggests the presence of alterations in prostanoid release in this vascular bed.

One of the main vasodilator prostanoid present in cerebral arteries is PGI (Andresen et al., 2006; Xavier et al., 2010; Peterson et al., 2011; Maccarrone et al., 2017). We observed an increase in BK-induced 6-keto PGF1 α (the stable PGI_2_ metabolite) release in cerebral arteries from MHC animals, similarly to that reported in aorta and mesenteric resistance arteries in diverse liver pathologies (González-Correa et al., 1996; Blanco-Rivero et al., 2009; Xavier et al., 2010). Since no differences in PGI_2_ synthase expression were observed, we could attribute the increased PGI_2_ release in MHC cerebral arteries to the augmented COX-2 expression, but we cannot exclude a possible enhancement in PGI_2_S activity.

Aside from vasodilator PGI_2_, COX-derived vasoconstrictor TXA_2_ also has a relevant role in the regulation of vascular tone in cerebral arteries (Hou et al., 2000; Andresen et al., 2006; Peterson et al., 2011). In fact, inter-relations between both prostanoids have been described (Cheng et al., 2002; Mollace et al., 2005). Modifications in TXA_2_ participation have been described in cerebral vasculature in different pathologies (Andresen et al., 2006). Regarding liver pathologies, both increases and decreases in TXA_2_ participation have been described in portal vein and splanchnic vasculature, respectively (Iwakiri and Groszmann, 2007; Gatta et al., 2008; Xavier et al., 2010), but, to the best of our knowledge, no reports regarding possible modifications in this vasoconstrictor factor have been observed in cerebral vessels. When measuring the stable TXA_2_ metabolite, TXB_2_, we found no differences between its release in cerebral arteries from SO and MHC rats, agreeing with the observation that TXA_2_ synthase expression was similar in both experimental groups. Altogether, these data show augmented PGI_2_ release in cerebral arteries from MHC rats, mainly due to the increase in COX-2 expression, while no modification in TXA_2_ release was observed.

An extravasation of ascitic fluid to the abdominal cavity is found in this MHC model after a six-week evolution of the pathology, being this symptom characteristic of ACLF. Regarding cerebral vasculature, the vasculopathy that we observed in the present study could be implicated in the development of HE related to an ACLF, characterized by a brain edema and hypoxia situation, which might consequently lead to CNS ischemia (Faraci and Heistad, 1990; Theodorakis et al., 2003; Sawhney et al., 2016). In fact, after a longer evolution of MHC (8 weeks or more), the animals die after going into a coma. Therefore, it would be interesting in the future to determine the pathogenic influence of vasoactive mediators in the development of the endothelial permeability that causes the HE related to ACLF.

In conclusion, we observed an enhanced BK-induced vasodilation observed in MCA from MHC rats, due to increased NO and PGI_2_. This augmented vasodilation might collaborate to increase brain blood flow in HE, and consequently be implicated in the brain alterations observed in ACLF.

## Data Availability Statement

The raw data supporting the conclusions of this article will be made available by the authors, without undue reservation.

## Ethics Statement

The animal study was reviewed and approved by All experimental procedures were approved by the Ethical Committee of the Universidad Autónoma de Madrid, and the Comunidad de Madrid.

## Author Contributions

LC, ES, MC, RR-D, and AG-R performed the experiments and statistical analyses and the systolic blood pressure measurements. IP and CN performed some experimental procedures, the surgical techniques, and the portal pressure measurements. IP, MA, and JA collaborated in the discussion of the results and the writing of the manuscript. MS and JB-R performed some experiments and statistical analyses, discussed the results, and wrote this manuscript. All authors contributed to the article and approved the submitted version.

## Conflict of Interest

The authors declare that the research was conducted in the absence of any commercial or financial relationships that could be construed as a potential conflict of interest.
